# The value of a healthy relationship

**DOI:** 10.7554/eLife.25412

**Published:** 2017-03-15

**Authors:** Bridget M Kuehn

**Keywords:** plain-language summaries, scientific publishing, public engagement, medical charities, patient groups

## Abstract

Biomedical science benefits when plain language allows patients to engage with all stages of the research process.

Huntington's disease is a degenerative disorder of the nervous system that develops in adulthood and can cause a wide range of symptoms. Children whose parents have Huntington's disease have a 50% chance of developing the disorder, and there is currently no way to prevent or slow it. The effect on families can be "cruel and unremitting" says Ed Wild, a researcher and clinician at the Institute of Neurology at University College London. However, a number of potential treatments are currently undergoing clinical trials, and this offers a glimmer of hope to families affected by the disease.

"Knowing about the latest research is something that can provide genuine hope, motivation, and the positivity they need to carry on in the face of this terrible thing happening in their family," says Wild, who is also one of the editors-in-chief of HDBuzz, a website that publishes plain-language reports on research into Huntington's disease.

HDBuzz was set up in 2011 by Wild and Jeff Carroll, an assistant professor of neurobiology at Western Washington University in Bellingham who comes from a family affected by Huntington's disease. "There was a real hunger for news that people could understand," recalls Wild. HDBuzz, which now receives hundreds of thousands of visitors each month, is one of a growing number of examples of efforts to make research accessible to the public and to help people to engage with clinicians and researchers more effectively.

**Figure 1:**
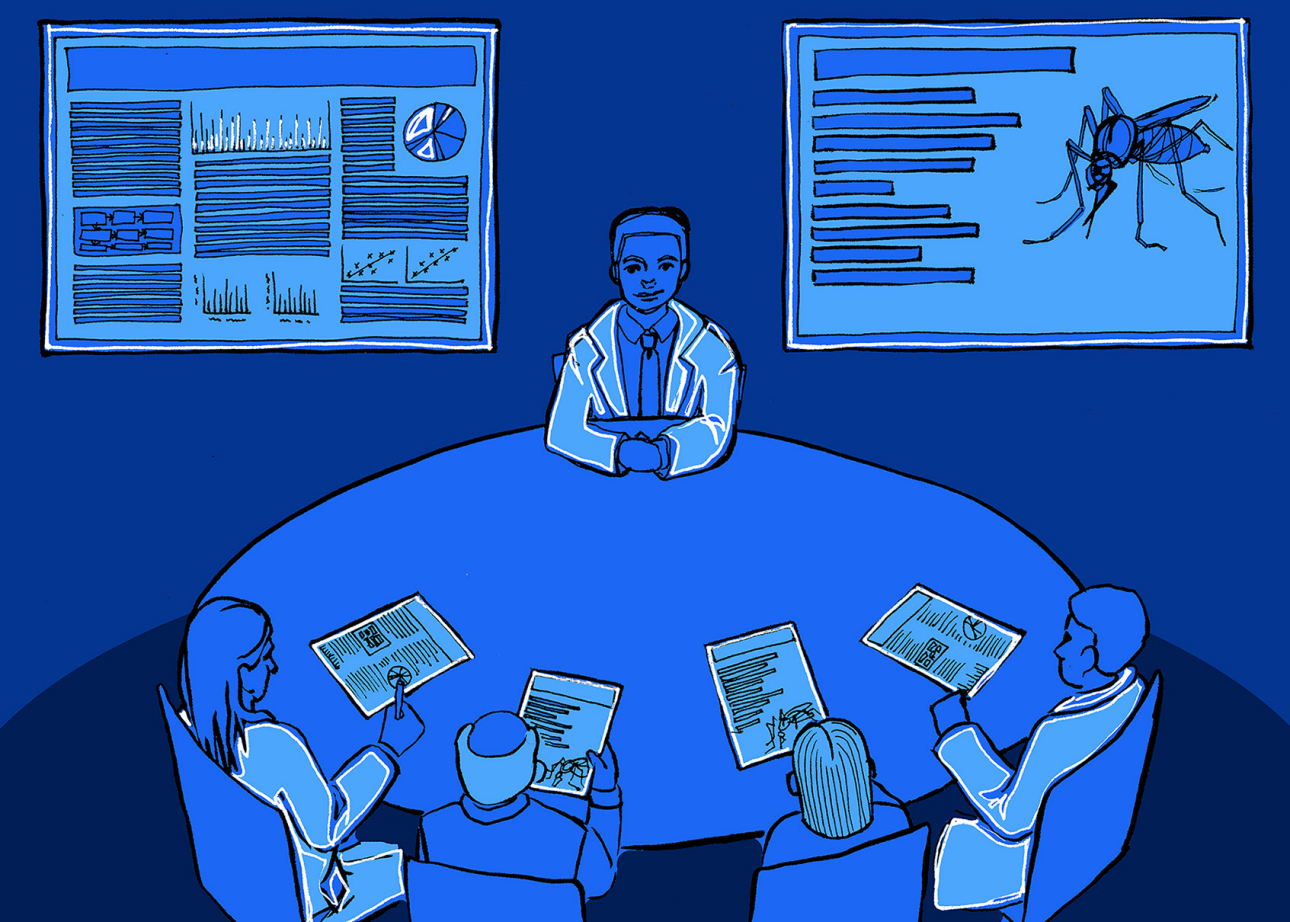
A number of medical research charities have patient representatives on their grant-evaluating committees and require grant applicants to include plain-language summaries with their applications. IMAGE CREDIT: vividbiology.com

## Public demand

The public has voracious appetite for health-related information, and patients and those who care for them can be very thorough in their online quests for information. Inspire, a US company that hosts more than 200 disease-specific social networks for patients and caregivers, has almost one million members. When surveyed about how they sought information related to health, respondents reported using an average of four sources. The top sources were condition-specific websites or blogs (77%), search engines (74%), medical and scientific articles (53%), and online support communities (47%).

"There is a myth that patients aren’t interested in medical journals," says Kathryn Ticknor, a linguist and senior research manager at Inspire. Indeed, Ticknor has found that patients' comfort with technical and medical language increases over time.

Wild agrees, and even knows patients and caregivers who regularly attend scientific meetings. "There is nothing to stop a family member from learning to understand a scientific article start to finish," he says. However, unlike researchers or clinicians in the field, such readers usually lack the context to know what the results mean for them.

The structure of a typical scientific article, which often focuses on what the findings mean for science, can also make it harder for unfamiliar readers to find the information they are looking for. "From a lay perspective, we want the bottom line," says Ticknor. "People want to know 'what does it mean for me?'"

Articles in the press may get to the point more quickly, but they often also lack the relevant context. Patients frequently ask Lester du Perron, a general practitioner in Amsterdam, about newspaper articles describing new therapies. Sometimes the medication mentioned in the article has only been tested in mice or is only intended for a specific subset of patients. These conversations can provide patients with all-important context, says du Perron, but they have limited reach. "When physicians counsel patients one-on-one it stays in that room," he explains. "You don’t get to educate all patients affected."

To reach a broader audience, du Perron and his colleague Tijs Stehmann, a physician and statistician, started Dokter Media, a website that provides expert reviews of medical news stories. Like HDBuzz, Dokter Media relies on scientific or clinical experts to write its articles.

## Engaged patients

In addition to making published research more accessible to the public, plain-language summaries are used to engage patients at all stages of the research process. Cancer Research UK and Muscular Dystrophy UK are among several medical research charities that require investigators applying for grants to provide plain-language summaries of their studies for the patient representatives that sit on its grant committees.

"Patient involvement in research review has become well established at Cancer Research UK," explains Jenni Macdougall, head of clinical research funding at the charity. While the research and clinical experts on the panel review the scientific merit of the proposed research, the patient representatives focus on whether the questions being asked matter to patients. They also assess the role of patients in study design, and the scientists’ plans for informing participants about results. "When patients are involved, you can address questions they have at an early stage," says Macdougall.

Patient representatives have also helped shape the grant review process at other organizations. For example, at least two people affected by multiple sclerosis serve on each grant-reviewing panel for the Multiple Sclerosis Society of Canada. These community representatives serve a limited term on the review committees to allow more to participate. But many continue to contribute by giving presentations at meetings or recruiting new representatives. "The goal long-term is to keep them engaged," says Angelica Asis, manager of research information and partnerships at the society. "They are almost like ambassadors for us." Like Cancer Research UK and Muscular Dystrophy UK, the society also posts the plain-language summaries for successful grant applications on its website.

Ultimately, the goal of these efforts is to give patients and their families a voice in shaping the science that may affect their lives. "It comes back to the idea of involving the people we are here to support in the decisions we are making," says Alison Stevenson, senior grants manager at Muscular Dystrophy UK.

## Accessibility

Meeting the demand for high quality, accurate plain-language summaries can be time consuming and labour intensive since it requires a mix of scientific knowledge and writing skills, and often involves multiple levels of review to ensure that articles are free from jargon and understandable by the public "on their own terms" (to quote from the HDBuzz website).

"It’s easy to forget that some words can be used differently by lay audiences and scientists," says Kathryn Ticknor. For example, adds Wild, for most people the word "expression" refers to a smile or a frown, but scientists use it regularly to describe gene expression. Early on, Wild enlisted patients and family members to consult about key phrases. Now, after having written lots of articles for HDBuzz, he believes that he and his expert writers "have a feeling for phrases that are potential pitfalls."

Writing plain-language summaries can also help scientists to focus on their study’s impact for patients.

Du Perron and Stehmann studied other plain-language initiatives before they got started on Dokter Media. They also recruited physicians-in-training from Maastricht University to learn about lay writing and to contribute to the site. "It’s still one of the things we are working on and plan to improve," he explains.

Cancer Research UK provides guidelines on what scientists should include in the summaries needed in their grant applications. They also periodically support workshops that offer training for young investigators, says Macdougall. During the training, a panel of patient representatives provides feedback and helps the researchers develop their communication skills.

"It may not always be the easiest thing for scientists to translate technical information into lay language," says Stevenson, "but increasing engagement with supporters means that the skill of communicating in lay terms is becoming more familiar and better practiced by researchers."

Writing plain-language summaries can also help scientists to focus on their study’s impact for patients. "They tend to write in their niche language, and they may forget to look at the big picture," says Asis. "Writing lay summaries helps them better communicate their research and extract meaning from the work."

The charities also provide large volumes of information for the public on their websites. To maintain its 8,000 pages of patient content and its clinical trials database, for example, Cancer Research UK employs a team of 15 nurse writers and health experts. The website also has Information Standard certification from the National Health Service, and was awarded the Crystal Mark by the Plain English Campaign. "Both of these standards means that patients can have trust that the information is of high quality, is evidence-based, easy-to-read, and that it is really focused on their needs," says Julie Sharp, head of health and patient information at Cancer Research UK. "We want to help people understand their situation and get their questions answered."

To ensure a high quality of content, many organizations put their plain-language information through one or two layers of editing and review by experts or patients. HDBuzz has also invested in an electronic editing system that manages the flow of articles, and it also pays its writers because, as Wild explains, paid writers often deliver their articles faster.

To pay these expenses, HDBuzz receives funding from several organizations with an interest in Huntington's disease. Each organization contributes less than they used to pay for less reliable writing, says Wild, and all the funding organizations agree that HDBuzz pays for itself in attracting donations. Other organizations like Dokter Media rely solely on volunteers and distribute their content primarily through Twitter and Facebook. Volunteers receive their reward in the form of positive feedback from both lay people and experts, says du Perron.

Plain-language summaries also help to bolster public support for the research enterprise and for organizations that support science and improved patient care. As Angelica Asis of the Multiple Sclerosis Society of Canada puts it: "If you don’t make that lay summary available, they may lose interest [in research]."

